# Community led total sanitation for community based disaster risk reduction: A case for non-input humanitarian relief

**DOI:** 10.4102/jamba.v8i2.183

**Published:** 2016-01-13

**Authors:** Daniel H. Mlenga, Yemane A. Baraki

**Affiliations:** 1 Disaster Management Training and Education Centre for Africa, Natural and Agricultural Sciences, University of the Free State, South Africa; 2 International Relief and Development, Mbabane, Swaziland

## Abstract

Sanitation related diseases have become endemic in southern Africa resulting in increased sanitation and hygiene morbidity and mortality. The region has experienced 318 400 cases of cholera and diarrhoea outbreaks between 2006 and 2012. There is insufficient financing for sanitation and hygiene activities, as people lack basic sanitation services, they engage in open defecation, the primary cause of faecal oral disease transmission. This study investigated Community Led Total Sanitation (CLTS), subsidy free, community based disaster risk reduction approach, for open defecation reduction, in four constituencies in Swaziland. Data collected from households, through a knowledge, attitudes and practices (KAP) survey illustrated that with appropriate training, involvement of traditional and community leaders, CLTS minimises open defecation. There is need of participatory rural appraisal through regular community monitoring and feedback meetings, as the disgust generated especially for women and youth, through the meetings, as well as group dynamics, steer the sustained construction and use of sanitation facilities. Lack of coordination between Non-Governmental Organisations (NGOs) leads to slow improvement of sanitation coverage, wherein the same communities are promoting CLTS and others are promoting Subsidy Based Sanitation Intervention (SBSI) which involves subsidies. It is recommended that there be coordination between partners for harmonisation of messages and an integration of the CLTS and SBSI approaches.

## Introduction

**FIGURE 1:**
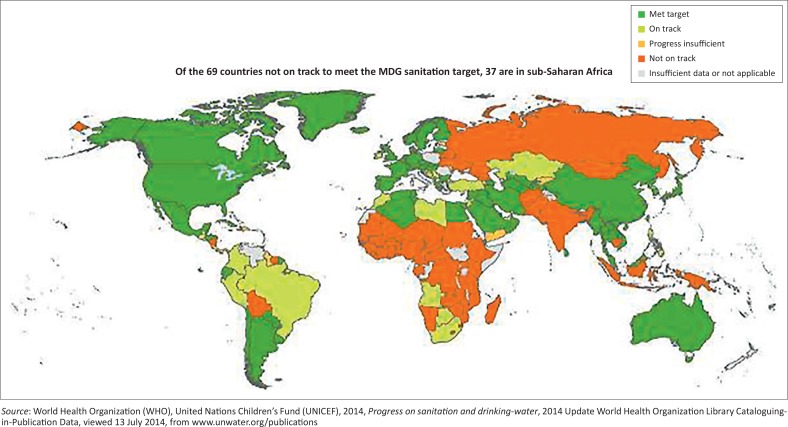
Progress towards Millennium Development Goals target.

Inadequate water and sanitation amenities and unhygienic habits play a part in millions of deaths of children annually, with almost 1.5 million children under five dying from diarrhoea each year (United Nations Children’s Fund [UNICEF] 2009). Clean portable water and good sanitation, which are universal needs and basic human rights, are indispensable foundations of poverty reduction and general human advancement. Without proper health and sanitation facilities to safely control the disposal of human faeces, the principal source of diarrheal pathogens, the health and sustainability of communities are compromised. Sanitation has, therefore, been incorporated by the United Nations into the Millennium Development Goals (MDG) with the MDG for sanitation lagging behind for most African countries (Figure 1), which in turn affects the delivery of other MDGs.

The unrelenting struggle against sanitation and water poverty in southern Africa continues to be the daily reality (Water Aid 2014). Sanitation related diseases have become endemic, and result in increased sanitation and hygiene morbidity and mortality. Child Health Epidemiology Reference Group (CHERG) (2012) estimated that two thirds of the region’s population, of 174 million people, lacks access to sanitation, whereas 100 million people lack safe water. The region has experienced cholera and diarrhoea outbreaks, with 318 400 cases reported between 2006 and 2012. In 2008 alone, over 167 000 cases and 4900 deaths were reported in nine countries.

### Sanitation coverage in Swaziland

**FIGURE 2:**
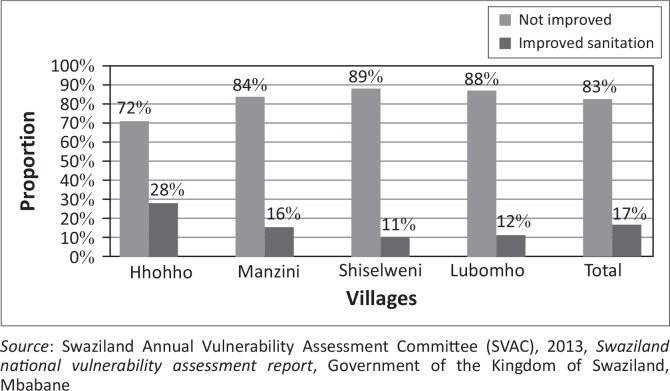
Proportion of household with improved sanitation.

Lack of adequate basic sanitation services often results in some people engaging in open defecation (hereafter: OD), the primary cause of faecal oral transmission of disease. Open defecation remains one of the most serious environmental threats to public health (Water and Sanitation Program [WSP] 2007). Swaziland Annual Vulnerability Assessment Committee (SVAC 2013) reported that an average of 83% of households do not have access to improved sanitation (Figure 2).

### Community based disaster risk reduction

The financial challenges affecting donor agencies and national government, and the sanitation challenges observed in Southern African Development Community (SADC) and Swaziland in particular, present a need to embrace low cost community based disaster risk reduction for the hazards and disasters that could be mitigated at community level. Community-based disaster risk reduction is a process initiated by the community and for the community, where hazard and risk management solutions are coming from the community itself. It is common knowledge that hazards and shocks are part of everyday life, exposed people living in vulnerable situations are not necessarily helpless victims waiting for unavoidable disaster to strike. Communities, as a first response implement community based disaster risk reduction initiatives which are founded upon the common participatory approaches. One bottom up, and cost effective approach, to reduce sanitation related hazards is Community Led Total Sanitation. The first crucial steps of community-based disaster risk reduction initiatives include awareness of the causal factors of disaster, followed by community strategic risk reduction planning aimed at identifying and countering risks linked with a particular hazard (United Nations Office for Disaster Risk Reduction [UNISDR] 2006b). These steps are consistent with those of Community Led Total Sanitation (CLTS) which are based on the concept of self-respect, by emphasising community dynamics coupled with individual perceptions and emotions as the drivers of sanitation provision by communities themselves (Harvey 2011).

### Community Led Total Sanitation

CLTS is a ‘zero subsidy’ innovative and inclusive approach that involves communities in ending OD, and is in partnership with government, Non-Governmental Organisations and other stakeholders. The approach facilitates rural communities to direct their own appraisal of sanitation challenges, come up with their own solutions and institute community-wide engagement with the fundamental aim of achieving Open Defecation Free communities. Unlike other approaches that are top down and command communities, this approaches lets communities make their own decisions. Emphasis is on empowering local communities to stop OD and to build and use latrines without the support of any external hardware subsidy (Kar & Pasteur 2005).

### Research objectives

There is a need for development partners and governments to embrace the notion that community initiated activities are being increasingly adopted, and have a higher level of sustainability, with the transformation from top down to participatory and community based programming. In many instances no subsidy strategies have been observed to result in increased adoption of technologies at community level, than the conventional subsidised approaches that prevail in low-income countries, whilst at the same time they promote self-sufficiency rather than dependency. This study investigated CLTS, as a community based disaster risk reduction approach, for OD reduction, with the end objective being to scale up LTS to increase sanitation coverage with minimum resources. In this process it embraced the following:

- the Hyogo Framework for Action 2005–2015
- building the Resilience of Nations and Communities to Disasters UN, 2005
- priorities for Action 1, 3 and 4 (UNISDR 2006b).

## Research methodology

**FIGURE 3:**
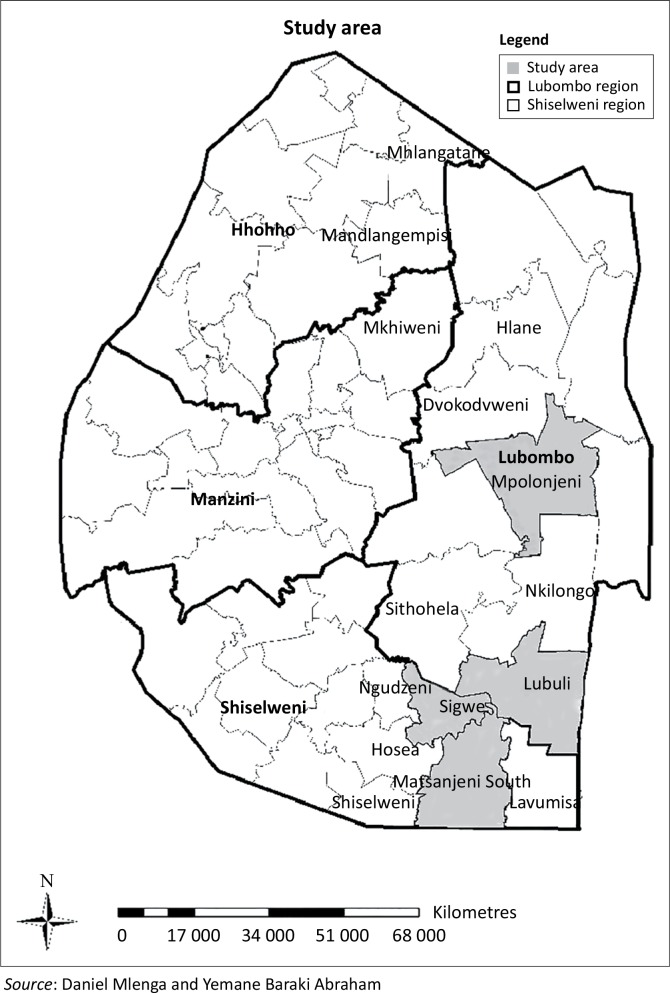
Map of study area.

The study was conducted in the Shiselweni and Lubombo regions of Swaziland in four of the six constituencies (Matsanjeni, Lubuli, Somntongo, Sigwe, Mpolonjeni and Dvokodvweni), where sanitation and hygiene programs were implemented by International Relief and Development between February 2013 and June 2014 (Figure 3). The sampling provided the opportunity to select, case and control households based on the communities exposed to Community Led Total Sanitation and Subsidy Based Sanitation Intervention (SBSI) by government and Non-Governmental Organisations between 2012 and 2013. The case-control study was designed to help ascertain if the community exposure to the CLTS campaign was associated with an improved sanitation coverage. The research approach applied quantitative and qualitative methodologies. The experimental design applied for the study was a cluster randomised design to assess the effect of the CLTS and SBSI on latrine adoption in four cluster communities.

Communities were selected using Ministry of Health sanitation coverage statistics, which were those identified to have had low sanitation coverage and were willing to be learning centres for the adoption and impact of CLTS. Through community mobilisation, in the targeted communities, households that had been exposed to the IRD facilitated CLTS campaign were identified, of which 100 were randomly selected for this study. Households in the same targeted community, that had been exposed to the SBSI by other development partners, were identified from community lists, in which 100 control households were also randomly selected. Trained survey enumerators collected data from the 200 households, through a knowledge, attitudes and practices (KAP) household survey.

The primary independent variables of interest for the study were sanitation coverage and household practices. Information was also collected on other sanitation indicators and demographic characteristics that could possibly be extraneous variables. Focus group discussions were conducted by the enumerators to assess sanitation and hygiene perceptions amongst groups. Key informant interviews were conducted with Rural Health Motivators and Non-Governmental Organisation (NGO) staff, who are the ‘trigger mechanisms’ utilised in the CLTS campaign, and are responsible in dissemination of sanitation information by government and other stakeholders. Observation was used to determine the type and status of a latrine at the household, based on a checklist that characterised the components of an improved latrine. Data collected through the KAP survey were analysed using SPSS 19.1, and compared communities involved in the cases, as well as case and control communities. Content analysis was applied to the information transcribed during focus group discussions.

## Results and discussion

### Community led total sanitation versus Subsidy Based Sanitation Intervention

The *Community Led Total Sanitation Campaign* was implemented through a collaborative effort between IRD community officers and community Rural Health Motivators. IRD, in collaboration with Ministry of Health Technicians, trained Rural Health Motivators to act as facilitators for CLTS. The trained facilitators visited selected communities, where they helped the communities analyse their areas and levels of open defecation, in an effort to generate a feeling of disgust for such practices and, at the same time, allowed the communities to decide on a plan of action. No subsidies were provided to motivate communities to improve their sanitation. The promotion of subsidies for sanitation improvement (*Subsidy Based Sanitation Intervention*) was conducted by government and various NGOs. Vulnerable households lacking improved sanitation infrastructure were selected through community based targeting mechanisms. Selected beneficiaries were trained on latrine construction and were supported with inputs that included cement, roofing sheets, vent pipes, toilet slabs and doors.

### Pre and post-Community Led Total Sanitation sanitation coverage

**FIGURE 4:**
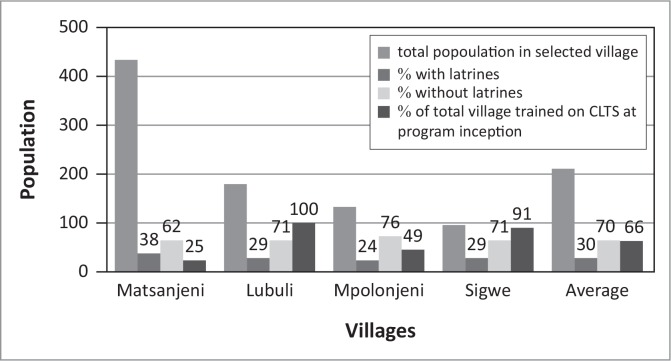
Status of selected villages at project inception.

At project inception 70% of case community members (Figure 4) did not have latrines and were practicing OD. Concerning the community members, 66% participated in CLTS sensitisation between February and August 2013. All members in the community were encouraged to participate in the CLTS sensitisation, regardless of whether you had a latrine or not.

**FIGURE 5:**
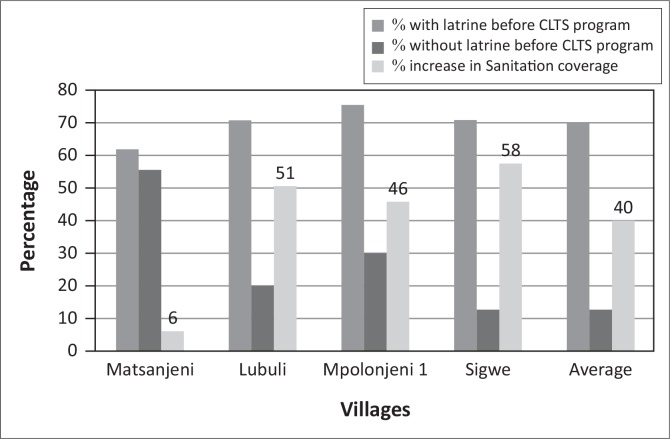
Change in sanitation coverage of selected villages after Community Led Total Sanitation intervention.

**FIGURE 6:**
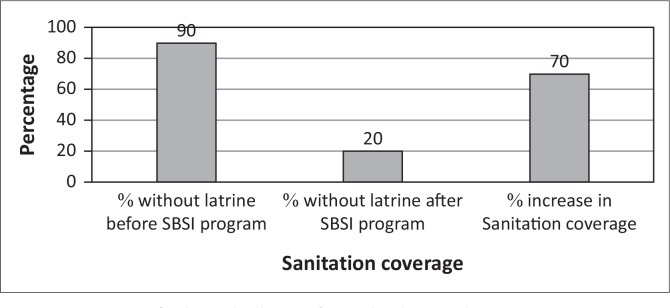
Status of selected villages after Subsidy Based Sanitation Intervention.

After the project commencement, within 10–16 months, there was a reduction of 40% in the number of people without latrines (Figure 5). In the same period, when analysing a control community, Mpolonjeni, where communities were exposed to SBSI, and were provided with hardware subsidy (slabs, vent pipes, cement and seats), the number of people with latrines stood at 90% (Figure 6) which was an increase in sanitation coverage of 70%. However, 40% of these latrines were missing, vents pipes, proper slab, roofing and doors. Therefore, the percentage of respondents with fully functional latrines (*where all provided inputs were* *utilised*), after SBSI, was 54%. This, therefore, showed that provision of hardware subsidies did not necessarily result in their total use. Some materials ended up being used for other household construction needs or were never used at all.

There were observed differences in the rate of change in sanitation coverage amongst the different case communities, with Sigwe having the highest change of 58% against an average of 40%, whereas Matsanjeni had the lowest percent sanitation change (Figure 6).

These differences could be attributed to the efficiency of the facilitators, as they were different facilitators who were used for the different constituencies. For sanitation to improve, CLTS demands excellent facilitation skills to ‘capture the moment’ when the entire community is triggered to act (WSP 2010). The role of the traditional leaders cannot be ignored as they are responsible for monitoring progress as well as encouraging village residents to construct and use latrines. It is also important to note that different village environments have different motivational drivers or levels of dissatisfaction (Jenkins & Curtis 2005). Therefore, for example, in Matsanjeni, where the CLTS uptake was low, an alternative approach should be investigated. The SBSI had a faster and higher sanitation coverage in the period under review, however, the increase in sanitation coverage came at a cost which is higher than that of CLTS.

### General socio-economic characteristics of the community

The socio-economic characteristics were analysed to determine their impact on latrine construction. Of the 200 respondents, 62% had latrines. The average household size was 9 people in which 51% of households had 6–10 members and 26% had less than 5 members. Despite the large household size, there was a high dependency ratio^1^ of over 70%, because most household members were incapacitated resulting from either sickness or school going children. The level of education of the respondents was high with only 36% having never attended any formal education, 34% having some primary education (Grade 1–6) and 34% having proceeded in schooling beyond primary school. There was no correlation, however, between household size and the level of education on the ability to construct latrine, the number of latrines constructed per household and the time when a latrine was constructed after the CLTS or SBSI sensitisation. The results are, however, not consistent with Admassu, Wubshet and Tilaye (2004) and Mukwaya and Kusiima (1998) where the researchers found that education of the household heads had a bearing on the availability of latrines in the homes. Household heads who had attended school were more likely to have latrines compared to those who had never attended school.

### Cost of Community Led Total Sanitation versus Subsidy Based Sanitation Intervention

**FIGURE 7:**
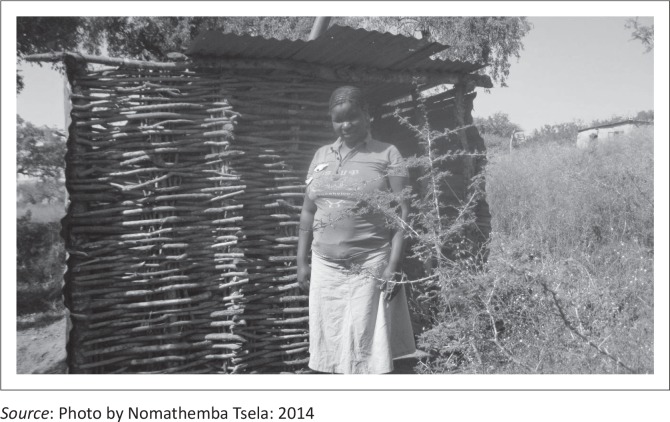
Latrine built after Community Led Total Sanitation sensitisation in Matsanjeni Constituency.

CLTS is community led with no hardware subsidy, but there is a cost involved. It costs 150 Emalangeni (*1 US$ = 10.5 Emalangeni*) per individual to train CLTS facilitators. In cases where NGO staff were facilitating the CLTS process it costs 90 Emalangeni per community member for a 2 day sensitisation process. Although there is a cost for the CLTS campaign by NGOs, the process is less costly than alternative options, because the costs incurred are inevitable program costs which will be incurred for any other development program implemented by NGOs. The average cost of latrines built without external subsidies averaged 300–1350 Emalangeni, in comparison to the cost of hardware subsidies which averaged 1500 Emalangeni for labour and material, CLTS was, therefore, cheaper. However, despite this advantage the survey revealed that there were differences in the types and quality of latrines constructed under CLTS and SBSI with all latrine under SBSI being pit latrines with slabs, whereas 69% of the latrines under CLTS did not have slabs and vent pipes. Many latrines in CLTS areas were constructed with local materials such as sticks and mud, wooden poles (see Figure 7), grass for walls and roofs, stones and other materials. Competing preferences and social acceptance of OD limits the construction and use of latrines rather than the inability to pay for them (Frias 2008). This, therefore, means that with the lower cost of latrines constructed under CLTS, if the household desires it, they can construct a low cost latrine.

**FIGURE 8:**
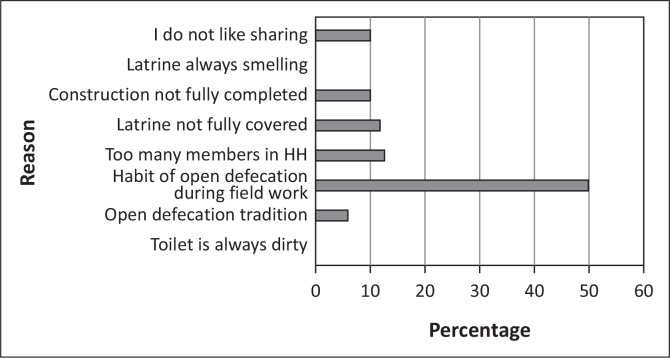
Reason for continued open defecation.

Whilst provision of subsidies increases sanitation coverage, with the number of people who continue to practice OD, it will be an investment of over 50 billion dollars, which is an estimate based on the current national sanitation coverage. Swaziland, like many other sub-Saharan countries, cannot afford to provide mass subsidies for sanitation improvement. There is no tangible evidence to support subsidy-driven programs to provide motivation for use of the sanitation facilities. Many studies, for example those in India (WSP 2007), show that people continue practising OD, not resulting from the lack of latrines, but because they see no reason to change their behaviour. Of the people who had latrines, 22% continued practicing open defecation. The main reasons attributed to this were spending too much time in the field (50% of the respondents), and having too many members in the household (13%) see Figure 8.

Community members continued to openly defecate when they are in their fields because of the distance to their homesteads. However, there is a cause for concern for those who continue to defecate openly as a result of not liking to share, the latrine not being fully covered and an open defecation tradition. There is no justification for the continuation of this OD, because they have a latrine, any minor challenge caused by the latrine should rectified. Therefore, ending OD is not just a matter of access to sanitation facilities, it also encompasses what motivates decision making such as household status, well-being, and situational goals (Jenkins & Curtis 2005).

### Factors influencing construction of latrines (those with latrines)

**FIGURE 9:**
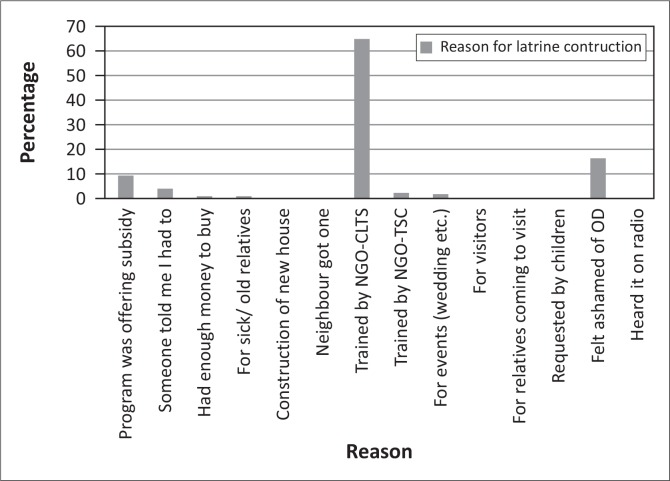
Reason for latrine construction.

To address the sanitation needs, it is important to know what influences the demand of latrines and their use (Jenkins & Curtis 2005). The study revealed that 66% of the respondents (Figure 9) decided to construct latrines after being sensitised by NGOs or Rural Health Motivators (RHM), 17% did so because they felt ashamed of OD, whereas 9% did so because they were provided with a hardware subsidy. When probed further, of those that were influenced by the CLTS campaign, 70% indicated that after the campaign they felt ashamed of OD practices, and as such they felt it was imperative that they changed their sanitation practices. This, therefore, emphasises the importance of the ‘triggering’ process, where a sense of shame and embarrassment is exhibited by those practicing OD. Harvey (2011) underlined that, there is not as strong an emotional trigger as that which results from CLTS.

Some communities were provided with a hardware subsidy. About these focus group discussions informed researchers that, although they were provided with the inputs, they did not have a reason to immediately construct latrines. This was because they were comfortable with the prevailing OD practices and this resulted in them constructing sub-standard latrines in which some of the provided materials were not used at all or used for other non-latrine purposes.

**FIGURE 10:**
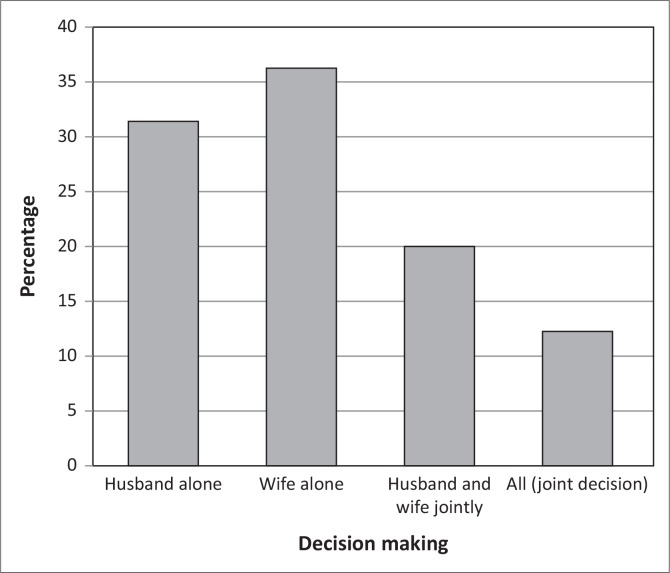
Who decides to build a latrine?

The study revealed that women (wives) played a huge role in the decision making about if and when to construct a latrine. With the need for labour and materials to construct the latrines, the husband’s contribution was also significant. Women alone made decisions, 36% of the time (Figure 10), on the need to construct a latrine, whereas 32% of the time, it was the husband alone who decided on the need to construct a latrine.

### Factors influencing construction of latrines (those without latrines)

**FIGURE 11:**
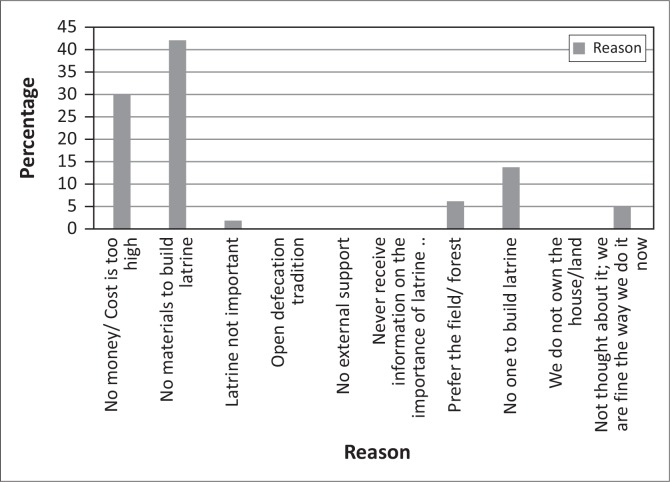
Reason for not constructing a latrine after Community Led Total Sanitation sensitisation.

Of the 38% of respondents who did not have latrines, 66% of had been sensitised on CLTS and 96% had discussed the need for latrines to reduce OD within their households. Of the respondents sensitised on CLTS 42% (Figure 11) indicated that the main reason why they did not construct their own latrines, despite being aware of the benefits, was a lack of construction materials, and 30% indicated a lack of money.

**FIGURE 12:**
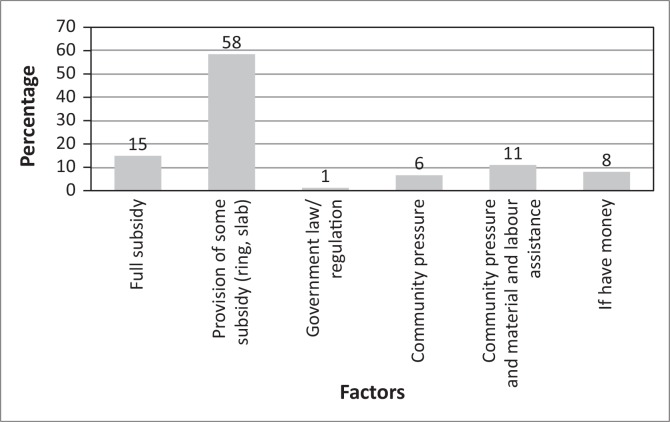
Required characteristics of a latrine.

Unsurprisingly, the main factor that could influence people to construct latrines (Figure 12) was 58% the provision of a hardware subsidy. When asked how and why it was possible for others in the same community, who had not received incentives, to construct latrines, 42% of the respondents highlighted that, the members who were able to construct latrines had received incentives secretly, and 32% noted that it was because of the intense community and traditional leadership pressure and feeling ashamed of OD.

From focus group discussions it was evident that within the same community there were conflicting approaches by NGOs and government. Some stakeholders were promoting CLTS whereas some were providing hardware subsidies. This, therefore, confused and demotivated communities, especially those that did not receive any incentive. In subsidy based, supply driven interventions, subsidising sanitation hardware has disadvantages because it destroys sanitation demand by diminishing user options and encouraging unwillingness to pay for sanitation by establishing a perception that sanitation should be partially or totally free (WSP 2005). In Bangladesh and India, where CLTS has had positive impacts, similar to Swaziland, in areas where hardware programs continue, CLTS is difficult, as communities instead of acting independently wait for assistance (Institute of Development Studies [IDS] 2009).

**FIGURE 13:**
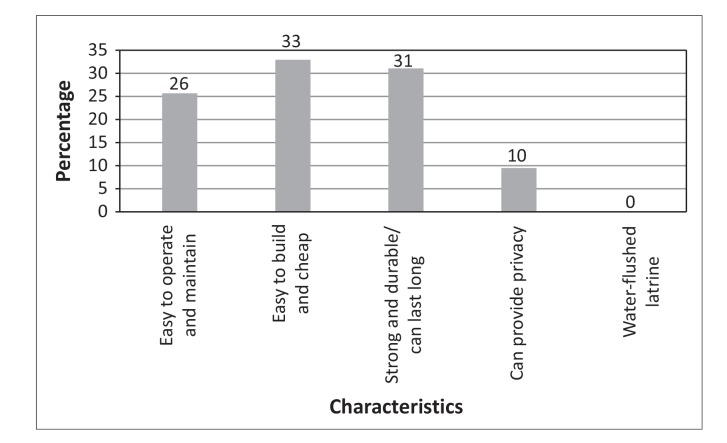
Required characteristics of a latrine.

It was noted that the type of latrine was not important, what mattered most was having a latrine that was cheap and easy to construct 33%, and easy to operate and maintain (26%) Figure 13. Provided there is availability of construction material households would construct a latrine.

Instead of providing free subsidies, what is also important is to provide people with information on where or how to access construction material. Concerning the respondents, 6% indicated that if they had access to loans, they would construct latrines. To further support this, 54% of the respondents indicated that it was possible to construct latrines without external support, however, there is a tendency of expecting to receive support, especially if the programs or projects are initiated by NGOs. There are conflicting priorities that tend to place latrine construction at the bottom of the main concerns of the household.

## Conclusion

The experience of implementing CLTS in Swaziland uncovered the possibility of increasing sanitation coverage and changing knowledge, attitudes and practices on sanitation and hygiene. The study revealed increases in sanitation coverage, thereby reducing the risk of faecal oral transmitted diseases through CLTS. With no external hardware subsidy, communities are made aware that the harm that results from OD is of their own making and change could be effected from within the household and community. Blanket and indiscriminate distribution of free inputs for all hazards could result in the development of a dependency syndrome, which could harm present and future disaster risk reduction initiatives.

Having awareness alone does not speed up the latrine construction, the triggering process should be complemented with monitoring and follow-up by the community health workers and traditional leaders encouraging, not forcing, the communities to change their sanitation practices. The community pressure itself contributes to further shame and embarrassment, thereby providing the need for a household to improve their sanitation status. The disgust generated, especially for women and youth, through the meetings as well as group dynamics, steers the sustained construction and use of sanitation facilities. By virtue of the process being community led and community based, it is more appreciated by the community and is, thus, more sustainable. This is a community led process and not something that is simply being imposed on them and, as such, they are willing to participate in the change process. Community dynamics have to be fully understood, as not all communities could be treated in the same way, and similarly a community cannot be treated differently from others. The lack of coordination and harmonisation of messages and approaches, between development stakeholders who are operating in the same community, leads to the slow adoption of development interventions as well as confusion amongst community members.

Decision making on the need to construct a latrine was reached by both men and women, with women taking the lead in realising the need to have a latrine at the homestead. To ensure that collaborative decision making is attained within the household, sanitation and hygiene interventions should involve the whole household, not just particular individuals in the household, thereby ensuring participatory decision making, construction and sustainable use of the latrine.

The level of education and household size did not influence the construction of latrines and it is critical, therefore, to identify other factors that influenced attitude change towards sanitation. As CLTS involved emotional sensitisation as well the influence of traditional leadership, the role of traditional influence is vital. Changing attitudes and practices is not an easy thing, as it requires time and effort. Having latrines within the homestead did not necessarily result in their use. The continued dissemination of sanitation and hygiene information through community agents, such as traditional leaders and Rural Health Motivators and in community meetings, will, overtime, result in the eradication of this practice. To complement this, sanitation and hygiene programs such as CLTS should involve all members of the household and not just women. Husbands and children should be involved to ensure that sanitation practices are changed more at household level than at individual level.

The SBSI had a faster and higher sanitation coverage in the period under review, however, the increase in sanitation coverage came at a cost which is higher than that of CLTS. If resources are available and there is a need for a fast and wide reach, SBSI may be considered as a preferred option. If resources are limited and fewer communities need to be reached, CLTS is relevant. It is critical, however, that the two approaches should not be applied to the same community.

## Footnotes

1 A measure of the portion of a population which is composed of dependents (people who are too young or too old to work).
